# A MULTIPRONGED STRATEGY FOR GROWING THE GERONTOLOGICAL WORKFORCE AT THE UNIVERSITY OF MASSACHUSETTS BOSTON

**DOI:** 10.1093/geroni/igae098.1618

**Published:** 2024-12-31

**Authors:** Edward Miller, Qian Song, Jeffrey Stokes, Ellen Birchander

**Affiliations:** University of Massachusetts Boston, Boston, Massachusetts, United States; University of Massachusetts Boston, Boston, Massachusetts, United States; University of Massachusetts Boston, Boston, Massachusetts, United States; University of Massachusetts Boston, Boston, Massachusetts, United States

## Abstract

The University of Massachusetts Boston is a public research university serving approximately 16,000 students. It is the most diverse university in New England and third most diverse university in the nation. The Gerontology Department is committed to growing the size and diversity of the workforce in aging services, policy, practice, and research. This presentation describes the multipronged approach taken to achieve this objective, while identifying associated facilitators and challenges. Departmentwide initiatives include prioritizing work in disparities in faculty hiring, implementing curriculum self-assessment and climate survey tools, and engaging in substantial marketing and outreach and collaborations with both internal and external constituencies. Doctoral program initiatives include expanding scholarship and training opportunities. Undergraduate program initiatives include changing the name of the major to “Aging Studies” to make the content clearer and more accessible, implementing a minor in Aging Studies to make it possible for students from across the University to earn a valuable credential, having all courses meet requirements in general education, and expanding the number and attractiveness of course offerings. The Department also implemented an initiative where undergraduates receive paid internships at local-area aging agencies with potential for continued employment post-graduation. Promoting diversity is closely connected to growing program enrollment with positive benefits for older adults and their families, caregivers, and communities.

